# Immune Profiles Identification by Vaccinomics After MVA Immunization in Randomized Clinical Study

**DOI:** 10.3389/fimmu.2020.586124

**Published:** 2020-11-10

**Authors:** Jorge Sanchez, Elena Gonçalves, Anuska Llano, Pedro Gonzáles, María Fernández-Maldonado, Annika Vogt, Angele Soria, Susana Perez, Samandhy Cedeño, Marco Antonio Fernández, Julien Nourikyan, Simon de Bernard, Carmela Ganoza, Eric Pedruzzi, Olivia Bonduelle, Beatriz Mothe, Carmen E. Gòmez, Mariano Esteban, Felipe Garcia, Javier R. Lama, Christian Brander, Behazine Combadiere

**Affiliations:** ^1^Centro de Investigaciones Tecnológicas, Biomedicas y Medioambientales, Universidad Nacional Mayor de San Marcos, Lima, Peru; ^2^Sorbonne Université, Inserm, Centre d’Immunologie et des Maladies Infectieuses (CIMIParis), Paris, France; ^3^IrsiCaixa AIDS Research Institute-HIVACAT, Hospital Universitari Germans Trias i Pujol, Barcelona, Spain; ^4^Asociacion Civil Impacta Salud y Educacion, Lima, Peru; ^5^Clinical Research Center for Hair and Skin Science, Department of Dermatology, Venerology and Allergy, Charité-Universitatsmedizin Berlin, corporate member of Freie Universitaet Berlin, Humboldt-Universitaet zu Berlin, and Berlin Institute of Health, Berlin, Germany; ^6^AP-HP Pitié-Salpêtrière, Paris, France; ^7^Flow Cytometry Facility, Germans Trias i Pujol Research Institute (IGTP), Hospital Universitari Germans Trias i Pujol, Barcelona, Spain; ^8^AltraBio, Lyon, France; ^9^Fundació Lluita contra la Sida, Infectious Diseases Department, Hospital Universitari Germans Trias i Pujol, Badalona, Spain; ^10^Centro Nacional de Biotecnología, Consejo Superior de Investigaciones Científicas (CSIC), Madrid, Spain; ^11^Infectious Diseases Department, Hospital Clínic, IDIBAPS, University of Barcelona, Barcelona, Spain; ^12^Faculty of Medicine, Universitat de Vic-Central de Catalunya (UVic-UCC), Vic, Spain; ^13^Institució Catalana de Recerca I Estudis Avançats (ICREA), Barcelona, Spain

**Keywords:** immunogenicity, cutaneous vaccination, intramuscular route, vaccinia virus vector, transcriptome

## Abstract

**Background:**

Our previous work has demonstrated the benefits of transcutaneous immunization in targeting Langerhans cells and preferentially inducing CD8 T-cell responses.

**Methods:**

In this randomized phase Ib clinical trial including 20 HIV uninfected volunteers, we compared the safety and immunogenicity of the MVA recombinant vaccine expressing HIV-B antigen (MVA-B) by transcutaneous and intramuscular routes. We hypothesized that the quality of innate and adaptive immunity differs according to the route of immunization and explored the quality of the vector vaccine-induced immune responses. We also investigated the early blood transcriptome and serum cytokine levels to identify innate events correlated with the strength and quality of adaptive immunity.

**Results:**

We demonstrate that MVA-B vaccine is safe by both routes, but that the quality and intensity of both innate and adaptive immunity differ significantly. Transcutaneous vaccination promoted CD8 responses in the absence of antibodies and slightly affected gene expression, involving mainly genes associated with metabolic pathways. Intramuscular vaccination, on the other hand, drove robust changes in the expression of genes involved in IL-6 and interferon signalling pathways, mainly those associated with humoral responses, and also some levels of CD8 response.

**Conclusion:**

Thus, vaccine delivery route perturbs early innate responses that shape the quality of adaptive immunity.

**Clinical Trial Registration:**

http://ClinicalTrials.gov, identifier PER-073-13.

## Introduction

Vaccines are usually injected into muscles or subcutaneous tissues. However, interest in skin vaccination (intradermal and epidermal application) has increased because the skin contains a relatively high number of resident antigen-presenting cells (APCs) that could drive immune responses to vaccination. The human epidermis is particularly rich in Langerhans cells (LCs), while dermal dendritic cells (dDCs) are found mainly in the dermis. Both types of cells can migrate to draining lymph nodes or to the spleen *via* lymphatic drainage. There, they initiate immune responses after interaction with antigen-specific T and B lymphocytes (1, 2). We have previously shown that the transcutaneous (t.c.) application of nanoparticles onto human skin explants can allow antigen to penetrate into hair follicles and thus target LCs. Our previous work highlighted the potential benefits of cyanoacrylate skin surface stripping for targeting hair follicles and thus its promise as a delivery technique for t.c. immunization (3). The involvement of LCs in CD8 T cell cross-priming suggests that vaccination *via* the t.c. route may be particularly useful in inducing T cell-mediated immune responses. A previous comparative clinical study using an inactivated influenza vaccine indicated that effector CD8+ T cells were preferentially amplified after t.c. application, compared with i.m. injection, which instead induced humoral responses (4). Vaccination through the hair follicle duct favors the targeting of LCs cells and has been shown to promote CD8+ T-cell responses in preclinical (5) and phase I–IIa (4, 6, 7) studies that used inactivated trivalent influenza vaccine. Previous studies have also shown effective DNA immunization *via* the hair follicle route in mice (5). In humans, we recently reported the safety and immunogenicity of a DNA vaccine administered by the t.c. + i.m. routes compared with the i.d. + i.m. routes; CD4 + and CD8+ cell-mediated immune responses were shaped differentially, with t.c. + i.m. responses showing a shift toward a more Th-17 dominated phenotype (8).

In addition, novel immune monitoring approaches applying transcriptomic analysis have enabled the development of hypotheses about the impact of vaccines on innate immunity. We recently applied these approaches to discover innate biomarkers able to predict the quality of immune responses to trivalent influenza vaccine (TIV) (4). Notably, blood gene expression and serum cytokine levels in the first few days after immunization were correlated with the intensity of the humoral response. These results provided important new insights into the molecular mechanism of vaccines and the choice of antigen, vector, and delivery route. The early up- or down-regulation of gene networks after vaccination was thus used to predict the intensity of immune response (months after vaccination) for different types of vaccines and administration routes (9). More hypotheses about the impact of routes of immunization on combined innate and adaptive immune signals need to be developed and tested.

Although vaccinia virus vaccines were historically administered *via* the skin (intradermal, i.d.), the version generally used today — modified vaccinia virus Ankara (MVA) — is administered by the i.m. route (10). Here we propose to investigate the t.c. and i.m. routes of immunization with an MVA-based HIV vaccine. Based on a thorough review of the literature on t.c. vaccination and our knowledge of this field, no evaluation of t.c. MVA vaccination has been documented in humans. Here, we used the MVA-B vaccine, a recombinant MVA vector expressing HIV-1 subtype B antigens (10, 11). This vaccine has been previously used in several clinical studies in HIV infected and uninfected individuals. All these studies administered MVA-B by intramuscular (i.m.) routes and generated long-lasting antigen-specific T-cell and antibody responses in 70–90% volunteers, thus consistently demonstrating the safety and immunogenicity of MVA-B vaccination by the i.m. route and positioning MVA-B as a vaccine candidate to explore alternative delivery routes (11–14).

We present here the results of the CUTHIVAC-003 trial, conducted in 20 HIV uninfected individuals in Lima, Peru. This Phase Ib randomized clinical trial aimed to examine the safety of recombinant MVA-B t.c. and i.m. delivery routes and to explore the resulting innate and adaptive immune responses. We specifically asked whether MVA-B vaccine administered by these two different routes of immunization: 1) is as safe by the t.c. as by the i.m. route, 2) produces the same or different qualities of adaptive immunity, and 3) induces different innate gene expression signatures, which may serve as indicators of late adaptive immune responses.

## Material and Methods

### Study Design, Approvals, and Participants

**Figure 1:**
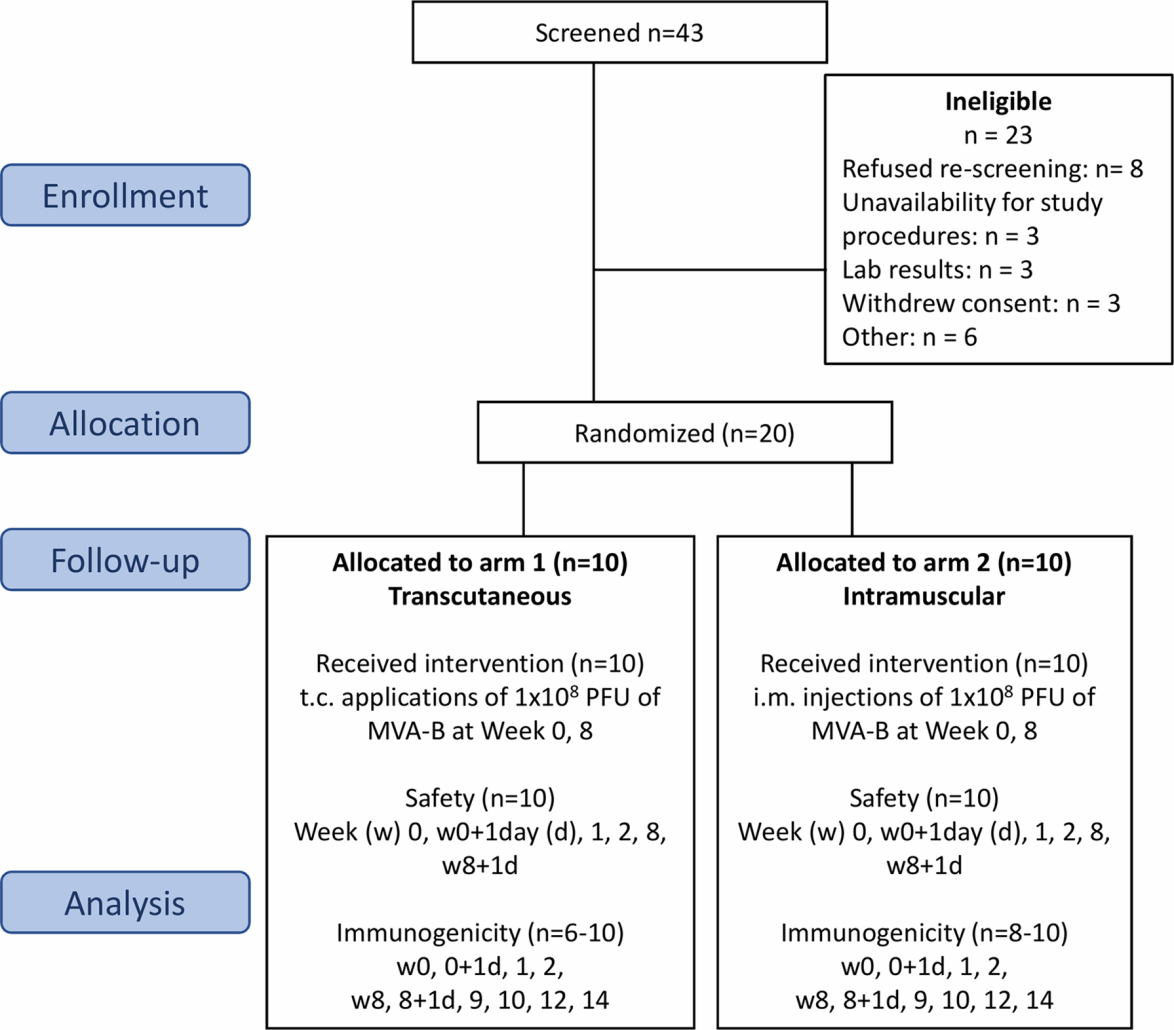
Flow chart of the successive steps of the MVA-B vaccine randomized phase Ib clinical trial. Flow of participants through the CUTHIVAC-003 clinical trial, according to Consolidated Standard of Reporting Trials (CONSORT). Twenty participants were enrolled, randomized in 2 arms (1:1) to receive MVA-B vaccine by the t.c. and i.m. routes on week (w)0 and w8. The primary end point was the safety of the t.c. and i.m. routes for MVA-B. Serum, PBMCs, and PAXgene samples were collected for exploratory analysis as secondary end points. Study approval number: IMPACTA IRB 0037-2014-CE; Peru NIH 396-2014-OG-OGITT-OPE/INS.

The CUTHIVAC-003 phase Ib randomized clinical study enrolled 20 volunteers aged 18–45 years at low risk of HIV infection, from October 15, 2014, to November 19, 2015. The participants received HIV-1 MVA Clade B vaccine by either t.c. or i.m. administration (1:1 ratio) after randomization for allocation at the Clinical trial unit (CTU) of the Asociacion Civil Impacta Salud y Educacion (IMPACTA) in Peru (**Figure 1**). Participants in the two groups had similar demographics (**Supplementary Table 1**) and both study arms contained equal numbers of men and women. They received their vaccine on day (d) 0 by the allocated route of administration to assess its safety and immunogenicity against the MVA vector alone and against MVA-B. The evaluations and procedures were conducted in accordance with the protocol, and the data collected were entered into electronic case report forms (eCRF). Eligible volunteers were randomized 24 h before the enrolment visit. This was an open trial so no unmasking was necessary. Block randomization of participants was performed centrally at the IMPACTA Peru CTU Data Management Center with a computer-generated algorithm and a manual backup procedure. The randomization was stratified by sex. This Phase Ib clinical trial is not intended to recruit a sufficient number of participants to have statistical confidence in the differences between groups. By the end of this study, 10 participants will have been exposed to each program in both groups, and this provides confidence around the 0–60% response/event ratios. Exclusion criteria were as follows: pregnancy (positive urine test), use of any topical treatment, tanning session in the last 4 weeks, presence of tattoos or surgical or traumatic metal implants, or excessive terminal hair growth on the investigational skin areas; skin-fold (cutaneous and subcutaneous tissue) of the upper right or left arm that exceeds 40 mm; previous vaccinia (smallpox) vaccination; HIV, hepatitis B or hepatitis C virus infections; Guillain-Barré syndrome; immunosuppressive treatment or other immunodeficiency; allergy to a vaccine component; medical history of skin cancer; acute infection or vaccination within 4 weeks of enrolment; body mass index below 19 or above 25; skin phototype V–VI; excessive terminal hair growth; and planned sun exposure 6 weeks before or during the study.

### Vaccine and Intervention

The MVA-B vaccine encodes a multi-HIV antigen, specifically a synthetic fusion protein comprising nearly complete protein sequences from the Gag, Pol, and Nef genes of the HIV-1 IIIB strain and the nearly complete protein encoding sequence from the Env gene obtained from the HIV BX08 strain (12). The MVA-B clinical lot was described previously (12). Both groups received 1.0 mL of the MVA-B preparation at 1 × 10^8^ PFUs (Plaque-Forming Units): in the t.c. group, 2 × 0.5 mL of the vaccine were applied to 2 pre-prepared skin sites on the upper arm (each site 16 cm^2^), and in the i.m. group, 1 × 1.0 mL of the vaccine was injected to the deltoid region of the nondominant arm. The dose to be used for each vaccination was the same as that previously given to HIV-infected and uninfected individuals in past clinical trials (12). The MVA-HIV Clade B vaccine has been used in several clinical studies (12, 15–17), and CUTHIVAC-003 was the fifth human experiment with this MVA vector expressing HIV-B antigens. Group A (t.c.) is the experimental arm; its participants received 2 doses (one prime and one boost) of MVA-B vaccine 8 weeks apart by a needle-free t.c. method for targeting hair follicles, according to the previously described standard operating procedure (6, 7). Briefly, the t.c. vaccination area (4 × 4 cm) was gently shaved, covered with a thin layer of cyanoacrylate (Superglue, UHU GmbH & Co. KG), and then stripped with adhesive tape to open hair follicles. The vaccine was applied to the stripped skin, inside a silicone barrier to limit the spread of the liquid, allowed to dry for 20 minutes, and then covered with a Comfeel adhesive bandage (Coloplast) for 24 h. Group B (i.m.) is the control arm. Participants allocated to this group also received 2 doses of MVA-B vaccine (one prime and one boost), 8 weeks apart by needle injection.

### Study Objectives

Safety was the primary study end point, defined by any local, laboratory, general or clinical adverse event (AE) of grade 3 or higher or any event of any degree that occurred in a participant who received at least one immunization and led to a clinical decision to discontinue immunization (**Figure 1**). The secondary end point was immunogenicity, assessed as the proportion of CD4+ and CD8+ T lymphocytes producing cytokines (IFNγ, MIP-1β, IL-2, and TNFα) in response to the vaccine. The amplitude of the humoral response was assessed by measuring neutralizing antibodies specific to the MVA vector in serum (NAb). Exploratory analyses used whole blood to study early innate gene expression and serum samples for cytokine levels.

### Safety Analysis

The hydrocolloid bandage was removed from the investigational sites 24 h after vaccine application. These sites were examined by medical professionals and local tolerance was assessed immediately after vaccination, and on d1, d7, and d14 after vaccination by evaluation for the presence of vaccine cutaneous adverse events (i.e. erythema or induration/swelling/edema) and axillary lymphadenopathy. On each visit, the volunteers were interviewed and asked about local and systemic symptoms including pain, tenderness, fever, malaise/fatigue, myalgia, headaches, nausea, vomiting, chills, arthralgia, and any cardiopulmonary symptoms. Information about AEs was requested on immunization day and up to the safety visit on the 14^th^ day after each immunization in a structured face-to-face interview. Participants were required to keep a daily diary card to record specific AEs that began within 14 days of the immunization, regardless of administration route. Each systemic AE was graded 0–5 (1, mild; 2, moderate; 3, severe, 4, life threatening; 5, death) and considered either “definitely related,” “probably related,” “possibly related,” “probably not related,” or “not related” to vaccine administration.

### MVA-eGFP Neutralization Assay

Anti-MVA neutralizing activity was evaluated by an assay that detected GFP by flow cytometry, in serum collected at week 0 (w0), w8, w9, and w12 (18, 19). This assay used HeLa cells as targets and a recombinant strain of MVA expressing the enhanced Aequoriae GFP. Serial dilutions of heat-inactivated serum were performed in 96-well round-bottom tissue culture plates (Corning) containing DMEM (Gibco, Invitrogen) supplemented with 2% fetal calf serum (FCS, Dominique Dutscher). MVAeGFP was then added to each well at a MOI of 0.25. After the plate was incubated for 1 hour at 37°C, 1 × 10^5^ HeLa cells were added. The incubation continued for 16 additional h at 37°C, 0.5% CO_2_. Cells were trypsinized and then washed with PBS supplemented with 0.5% FCS and 2 mM EDTA and fixed with 2% formaldehyde. GFP expression was analyzed with FACSCanto II and by using Diva software (BD Biosciences) and FlowJo software. The percentage of neutralization was defined as the reduction in the number of GFP-expressing cells compared to the number of GFP-expressing cells in untreated control wells.

### Quanterix Technology (Digital ELISA)

The Simoa™ (single molecule array) HD-1 analyzer was used for ultrasensitive multiplex immunodetection of cytokines; we used the single kits for human IL-1β (Simoa^®^ data sheet item 101605), CXCL10 (Simoa^®^ data sheet item 101132), and IL-8 (Simoa^®^ data sheet item 100198) and a 3-plex B kit for the simultaneous detection of TNFα, IL-6, and IL-17α (Simoa^®^ data sheet item 101319) in accordance with the manufacturer’s instructions. In this approach, antibodies to specific proteins were immobilized to color-encoded paramagnetic beads, the bead mixtures incubated with serum samples, and multiple specific proteins captured by the corresponding specific beads. Next, the enzyme-labeled beads were isolated, sealed in arrays of femtoliter-sized wells in the presence of enzyme substrate, and loaded into arrays on the Simoa™ microfluidic disk. Finally, the fluorescence generated by a single enzyme was detected by an uncooled CDD camera by using a white light excitation source to determine the concentration of each protein with high precision. We referred to standard curves obtained with recombinant calibrator proteins to calculate concentrations according to different calibration ranges: IL-8 (0–300 pg/L), IL-1β (0–120 pg/mL), CXCL10 (0–200 pg/mL), TNFα (0–28 pg/mL), IL-6 (0–15 pg/mL), and IL-17α (0–10 pg/mL).

### Autologous Antigen-Presenting Cell Generation (Autologous-APCs)

To generate autologous antigen-presenting cells (APCs), autologous peripheral blood mononuclear cells (PBMCs) from samples collected at baseline from 17 of the 20 participants were thawed and stained with a cell tracer (CFSE, Thermo Fisher Scientific), in accordance with the manufacturer’s instructions. Briefly, PBMCs were resuspended in phosphate-buffered saline (PBS) at a final concentration of 10 × 10^6^ cells/mL and labelled with CFSE at 1 µM for 6 minutes at room temperature in a roller mixer in dark conditions. The cells were then washed 3 times in PBS supplemented with 10% FCS and incubated for 30 min at 37°C, 5% CO_2_. After the incubation period, the CFSE-labelled cells were resuspended in RPMI medium containing 2% FCS and incubated overnight under 3 separate conditions: infected with 2 PFU/cell of the vaccine immunogen (MVA-B), infected with 2 PFU/cell of the vaccine vector alone (MVA), or not infected. The next morning, the autologous-APCs were resuspended at 2.5 × 10^6^ cell/mL in RPMI medium supplemented with 20% FCS and used as stimulus in the intracellular cytokine staining (ICS) assay. For the ICS, PBMCs sampled at pre-(w0) and post-vaccination time points (w2, w8, w10, w14, and w20), were thawed and resuspended at 2.5 × 10^6^ cell/mL in RPMI medium, supplemented with 20% of FCS, and used as effector cells. The ICS assay was set up by co-culturing the two cell types at a 1:2 ratio (Autologous-APCs: Effectors) in the presence of 1 µL/mL of the protein transport inhibitor Monensin (GolgiStop, BD Bioscience) and 0.7 µg/mL of anti-CD28 (BD Bioscience) during an overnight incubation at 37°C, 5% CO_2_. As positive controls for the assay, effector cells were cultured alone in the presence of either 1) anti-CD3/28 Dynabeads (Thermo Fisher Scientific) according to the manufacturer´s instructions, or 2) 10 ng/mL PMA (SIGMA) and 1 µM ionomycin (SIGMA).

### Intracellular Cytokine Staining (ICS) Assay

The anti-vaccine responses were detected by measuring effector cell cytokines in the ICS assay. Briefly, to exclude dead cells, monocytes and B cells, cells were stained with the Live/Dead fixable Violet Dead cell stain kit (Invitrogen), CD14-Pacific Blue, and CD19-V450 and gated in a dump channel as described (20). Additional surface markers were used to study the T-cell lineages (CD3, CD4, and CD8), T-cell activation (CD38 and HLADR), and maturation phenotypes (CD45RA and CCR7). The cells were fixed and permeabilized with the Cell Fixation and Cell Permeabilization Kit (Invitrogen) in accordance with the manufacturer´s guidelines, and intracellularly stained to detect effector cytokines (IFNγ, MIP-1β, IL-2, and TNFα). Cells were resuspended in PBS supplemented with 1% FCS and acquired on a Fortessa SORP flow cytometer (BD, Flow Cytometry Facility, IGTP). Details of the clones and manufacturers of the fluorochrome-conjugated antibodies are reported in **Supplementary Table 2**. Data were analyzed with FlowJo software, version 10.6. **Supplementary Figure 1B** presents the gating strategy and an example of row flow cytometry analysis. Total vaccine-specific T-cell responses were calculated by adding up the quadruple, triple, double and single-positive cells for the expression of the intracellular cytokines IFNγ, MIP-1β, IL-2, and TNFα. The frequencies of the cells producing each of the possible combinations were calculated with the Boolean gating function on the FlowJo software. Background cytokine responses detected in unstimulated negative controls were subtracted from those detected in vaccine-stimulated samples.

#### RNA Extraction and Data Preprocessing for Transcriptomic Analysis

Whole-blood samples of 2.5 mL were collected in PAXgene RNA (PreAnalytix) in tubes from each volunteer at baseline before the prime vaccination (w0) and after the first (w0+1, w1, w8), and second (booster) immunizations (w8+1, w9, w12) and stored at -80°C. Total RNA was extracted according to procedures set forth in the PAXgene blood RNA Kit (PreAnalytiX, Hombrechtikon, Switzerland) handbook. RNA purity and integrity were assessed on the Agilent 2100 Bioanalyzer with the RNA 6000 Nano LabChip reagent set (Agilent, Palo Alto, CA, USA). The RNA integrity number were between 7,9 and 9,7. RNA quality and quantity were similar in the two batches of samples and allowed hybridization of all samples using Affymetrix’ standard amplification protocol. Sample preparation for microarray hybridization was carried out as described in the Affymetrix GeneChip WT PLUS Reagent Kit User Manual (Affymetrix, Inc., Santa Clara, CA, USA). Hybridization (to Affymetrix Human Gene 2.1 ST Array Plates), washing, staining, and scanning took place in an Affymetrix GeneTitan system, controlled by the Affymetrix GeneChip Command Console software w4.2. Background signal correction was performed by applying the backgroundCorrect function from the limma package to the perfect match (PM) signals with R Software 3.3.1. The underlying model is the normal-exponential convolution model from RMA (21). The variance-stabilizing transformation algorithm (function justvsn from package vsn) (22) was applied to the background-corrected signal, and the signal then transformed back to its usual scale by exponentiation (base 2). To make the chips comparable, a quantile normalization (23) (function normalize from package affy) was then applied to the variance-stabilized signal. The probe signals for replicated arrays were averaged and quantile normalization performed again. In all, 24 768 probes were analyzed.

### Statistic and Graphic Representations

Statistical analyses of the immunological and transcriptomic data and graphic representations of the kinetics were performed with Prism 6.0 (GraphPad Software Inc.). The potential associations between serum cytokine levels or gene expression induced early after vaccination and the percentages of cytokines secreting CD8+ T cells or neutralizing antibody titers were evaluated by Spearman correlation, with significance defined by a *p*-value < 0.05. The heatmaps were performed with R with values centered and scaled in the row direction, Pearson’s correlation used as the distance method, and dendrograms computed and reordered based on row means. Ingenuity pathway analysis (IPA) and the open database Enrichr were used for functional enrichment analyses to identify new targets or candidate biomarkers within the context of biological systems. It provided the cell types, canonical pathways, molecular/cellular functions, and networks that were statistically overrepresented in the gene signatures. Transcriptomic data were analyzed with R software 3.3.1. All data were analyzed with mixed linear regression models. *P*-values were then corrected for multiple comparisons by using the Benjamini and Hochberg procedure to control the false discovery rate (FDR). Corrected *p*-values less than 0.05 were considered to indicate statistical significance from transcriptomic data analyses for comparisons between time points. The Volcano plot of the significantly differentially expressed genes was performed with R, based on the –log10 of the adjusted *p*-value and the log2 of fold-change.

#### Study Approval

The study was conducted in accordance with the Declaration of Helsinki and the International Conference on Harmonization Good Clinical Practice guidelines and approved by the relevant regulatory and independent ethics committees. Each participant provided written informed consent before study entry. The study was registered and approved by the Peru regulatory authorities (IMPACTA IRB 0037-2014-CE; Peru NIH 396-2014-OG-OGITT-OPE/INS).

#### Data Availability Statement

All data analyzed in this study are included in the published article. The normalized microarray data that support the finding of this study have been deposited in ArrayExpress with the accession code E-MTAB-9642.

## Results

### Study Protocol

In this study, 20 subjects randomized for route of administration received two immunizations of the MVA-HIV Clade B vaccine by their allocated routes on w0 and w8 from October 15, 2014, through November 19, 2015 (**Figure 1**). The vaccine was a recombinant MVA vector expressing HIV-1 antigens derived from the natural HIV-1 isolate BX08 (for gp120) and isolate HIV-IIIB (for Gag-Pol-Nef) (12). No subject was lost to follow-up. Subjects in both arms had similar demographics, and both groups included equal numbers of men and women (**Supplementary Table 1**). The primary study end-point was safety, assessed by comparing the number of local and systemic adverse events from MVA-B administration by the i.m. and t.c. routes respectively. The secondary end-point was immunogenicity, assessed by measuring the proportion of T lymphocytes that produce cytokines (IFNγ, MIP-1β, IL-2, and TNFα) in response to the MVA vector and to MVA-B. The amplitude of the humoral response was assessed by serum measurements of neutralizing antibodies specific to the MVA vector. Innate immunity was explored by measuring cytokine/chemokines in serum samples and whole-blood gene expression at indicated time points.

### MVA-B Was Safe in All Study Participants by Each Route of Administration

Safety was assessed by collecting the number of local Adverse Events (AEs) in the immediate area where the vaccine was administered as well as recording the systemic, laboratory, and clinical AEs (**Table 1**). We observed fewer local and systemic side effects after t.c. compared with i.m. vaccination, for the administration of both doses of vaccine. In the t.c.-vaccinated individuals, general side effects (fatigue, myalgia, and chills, five cases) were observed from w0+1d to w1 after the prime immunization and at d1 (fatigue, headache, and pruritus, four cases) after the boost (**Table 1**). All participants reported local side effects (tenderness and pain) after the i.m. administration. In addition, 36 separate incidents of systemic side effects at w0+1d, 14 cases at w1, and 43 cases at w8+1d were reported after i.m. administration. The systemic side effects following i.m. immunizations were mainly fatigue, myalgia, headache, chills, and arthralgia. These local and general side effects disappeared after a few days. The side effects related to vaccination accounted for the largest number of AEs, as observed previously (12). No serious AE was reported during the study. Overall, t.c. and i.m. MVA vaccinations were safe.

**Table 1:** Local and general adverse events induced after each injection of MVA-B, by route of administration.

SYSTEMIC AEs		Transcutaneous			Intramascular		

		Definitely Probably		Total	Definitely Probably		Total
First dose (w0)							
**d1**	Fatigue	2		2	6		6
	Myalgia	1		1	2		2
	Fever				2		2
	Headache				2		2
	Arthralgia				1		1
	Chills				1		1
	Common cold					1	1
	Nausea				1		1
	Vomiting				1		1
	**Total w0+1d**	**3**	**0**	**3**	**16**	**1**	**17**
**w1**	Fatigue				3		3
	Myalgia				2		2
	Fever				1		1
	Headache				2		2
	Chills	2		2	1		1
	**Total w1**	**2**	**0**	**2**	**9**	**0**	**9**
Second dose (w8)							
**d1**	Fatigue	1		1	8		8
	Myalgia				5		5
	Fever				2		2
	Headache		2	2	4		4
	Arthralgia				4		4
	Chills				4		4
	Nausea				2		2
	Vomiting				1		1
	Pruritus	1		1			
	**Total w8+1d**	**2**	**2**	**4**	**30**	**0**	**30**
**LOCAL AEs**							
		**Yes**	**No**	**Total**	**Yes**	**No**	**Total**
First dose (w0)							
**d1**	Pain				9		9
	Tenderness				10		10
	**Total w0+1d**	**0**	**0**	**0**	**19**		**19**
**w1**	Pain				4		4
	Tenderness				1		1
	**Total w1**	**0**	**0**	**0**	**5**	**0**	**5**
Second dose (w8)							
**d1**	Pain				5	1	6
	Tenderness				6	1	7
	**Total w8+1d**	**0**	**0**	**0**	**11**	**2**	**13**

Safety was evaluated by observation of local adverse events (AEs) in the area where the vaccine was administered and the systemic, laboratory, or clinical adverse events. The investigational sites were examined by medical professionals and local tolerance at the vaccine administration area was assessed on day 1 after vaccination by evaluation for erythema, pruritus, burning and desquamation (local reactions were yes vs no and size). On each visit, the volunteers were interviewed for systemic reactions including rashes, pain, fever, headaches, shivering, diarrhea, and malaise. Participants were required to keep a daily diary of requested AEs that began within 14 days of the immunization, regardless of administration route. Each AE was considered either “definitely related,” “probably related,” “possibly related,” “probably not related,” or “not related” to vaccine administration.

### Quality of Immune Response After i.m. and t.c. Administration

**Figure 2:**
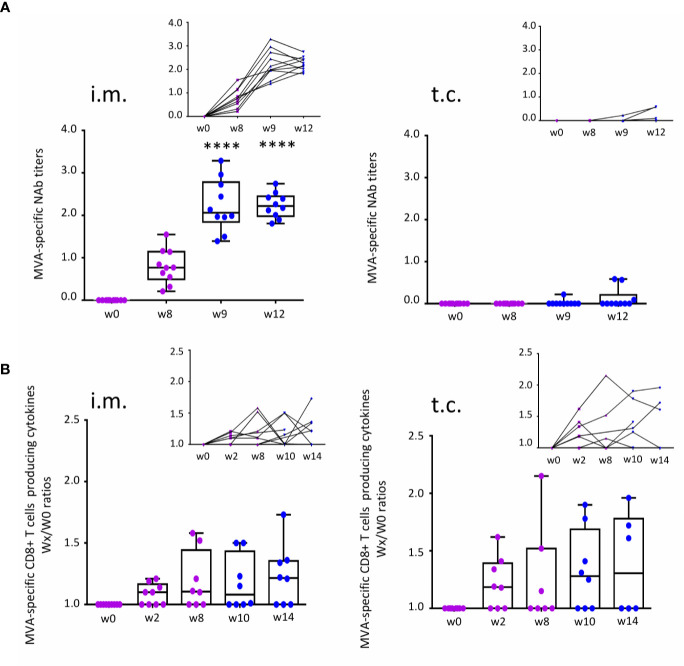
Measurement of immune responses to MVA-B vaccine after t.c. and i.m. vaccination. **(A)** MVA-specific neutralizing antibody (NAb) response before (w0) and after the prime (purple, w8) and boost (blue, w9 and w12) vaccinations for each administration route: i.m. (n = 10) and t.c. (n = 10). Data analysis presents neutralizing antibody titers (log 1/EC50) in box-and-whisker plots with the minimum to maximum showing all points (from the bottom up: the minimum 25th percentile Q1, median, 75th percentile Q3, and maximum values) and in line charts. **(B)** MVA-specific CD8+ T cells expressing intracellular cytokines IFNγ, MIP-1β, IL-2, and TNFα before (w0) and after prime (w2 and w8) and after the boost (w10 and w14) vaccinations for each administration route: i.m. (w0 and w2, n = 9; w8, w10, and w14, n = 8) and t.c. (w0, w2, and w10, n = 8; w8, n = 7; w14, n = 6). Data analysis presents the ratios wx/w0 of the percentages of MVA-specific CD8+ T cells (Boolean IFNγ, MIP-1β, IL-2, and/or TNFα). ANOVA Friedman test was applied (****p < 0.0001).

In the context of either DNA vaccine (8) or seasonal trivalent influenza (TIV) vaccination (4, 6, 7), we have previously shown that after t.c. immunization, CD8 T-cell responses occur only when there is no humoral response to DNA vaccine. Here, we aimed to analyse the quality and intensity of humoral immunity versus CD8 T-cell responses to a viral vector-based vaccine administered by a t.c or an i.m. route. The neutralization ability of MVA-specific titers (MVA-NAb) was measured by assays that neutralize MVA in-vitro infectivity. MVA-specific CD4+ and CD8+ effector T -cells producing single or multiple cytokines (IFNγ, MIP-1β, IL-2, and TNFα) were assessed by flow cytometry. The gating strategy for the ICS assay and MVA-B dot plots are presented in **Supplementary Figure 1**. We focus on the previously described, robust cellular immunity to the vaccine vector (MVA). Still, for a limited set of samples, the correlation between T cell responses to MVA and MVA-B was established (**Supplementary Figure 2**). **Figure 2** summarizes our observation of humoral and cellular immune responses according to MVA-NAb titers (log 1/EC50) and MVA-specific effector CD8 T cells (ratio of each time point relative to baseline at w0). As expected, the t.c. route did not induce MVA-NAb, while the participants vaccinated by the i.m. route showed a significant increase in MVA-NAb titers (*p* < 0.0001) at w8 and w9 (**Figure 2A**). This observation confirms our previous finding that targeting vaccines to the hair follicle (t.c.) did not favour humoral immunity (2, 6–8). Having previously shown that this route favors CD8 immune responses instead, we monitored the presence of MVA-specific CD8+ T cell populations over the study period. We found some increase in the number of MVA-specific cytokine-producing CD8+ T cells after both i.m. and t.c. vaccination compared with baseline in half the subjects (**Figure 2B**). The t.c. immunizations induced CD8 T-cell responses slightly earlier (w2) (**Figure 2B**, right panels) than the i.m. immunizations (w8) (**Figure 2B**, left panels). Of note, the MVA and MVA-B-specific CD8 T-cell responses had similar levels of intensity (**Supplementary Figure 2A**). We also measured CD4 T-cell responses in both study arms, however only a few individuals in each arm showed detectable responses (**Supplementary Figure 2B**). In conclusion, t.c. immunization favors early CD8 T-cell responses in the absence of MVA-specific NAb responses, while i.m. immunization induced strong NAb responses and late CD8 T-cell responses.

### Serum IL-6 and Related Genes Expressed at 1 Day After i.m. Immunization Correlated With MVA-NAb Responses

**Figure 3:**
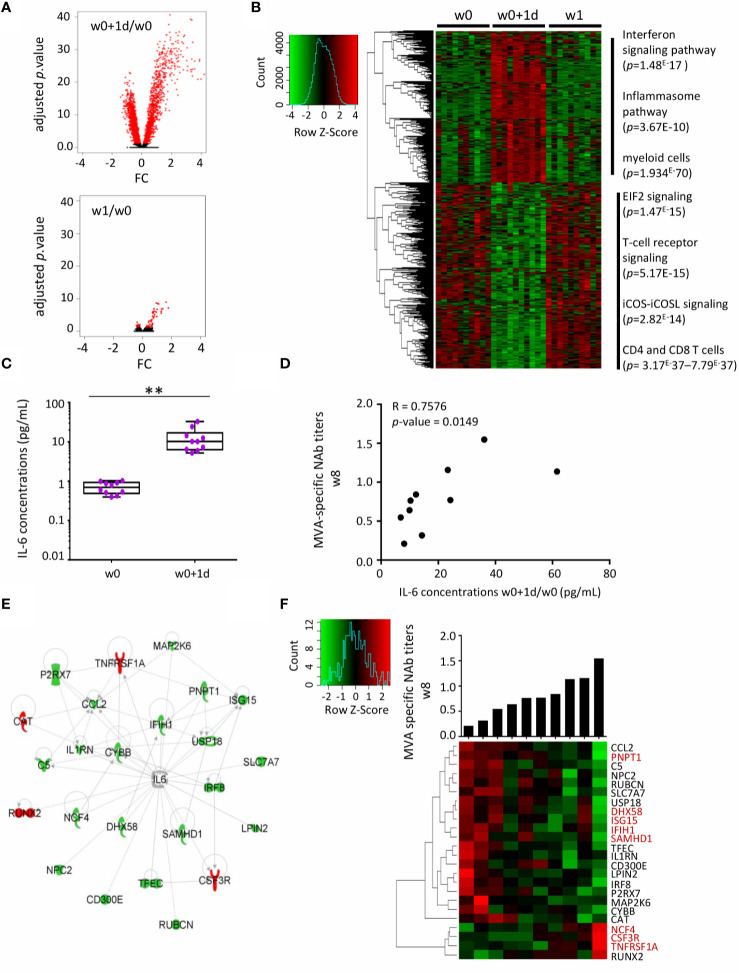
Serum IL-6 and related whole-blood genes one day after MVA-B vaccination related to MVA-Nab responses after i.m. immunization. **(A)** Volcano plots of the 2611 and 54 genes differentially expressed one day (w0+1d, upper panel) and one week (w1, lower panel), respectively, after MVA-B i.m. vaccination, compared with baseline (w0), according to the -log10(adjusted *p*-value) and log2FC. Significance is defined by an adjusted *p*-value < 0.05 (red). **(B)** The hierarchical clustering is based on the expression profiles of these significantly differently expressed genes (2611 + 54) at each of the three time points: w0 (n = 10), w0+1d (n = 10), and w1 (n = 10). The color-gradient from green (-4, low) to red (4, high) indicates gene expression levels (log2). The functional enrichment was performed with IPA software. *p*-values by IPA are indicated for each pathway. **(C)** Serum concentrations (pg/mL) of IL-6 at times w0 and w0+1d presented in box-and-whisker plots. The Wilcoxon test compares w0 to w0+1d, with a significant increase observed at w0+1d (**p < 0.01). **(D)** Correlation graph shows the significance of the difference between MVA-specific NAb titers at w8 (log1/EC50) and IL-6 serum concentrations at w0+1d/w0 (pg/mL). The Spearman test was applied, with significance defined by a *p*-value < 0.05. **(E)** IPA highest scored biological network for the 24 correlated genes (d1) involved in IL-6 signaling and correlated with MVA-Nab responses at w8. The downregulated genes in high responders are in green and the upregulated genes in red. **(F)** The hierarchical clustering is based on the fold-change expression profile (w0+1d/w0) of these 24 genes. The histogram shows MVA-NAb response intensity at w8 (log1/EC50) for each individual, from lower to higher responders. In red, genes in common with the literature (3, 8, 24).

We sought to define an early innate gene signature associated with the quality of immune responses by route of immunization. First, we analyzed the i.m. study arm, where both cellular and humoral immune responses were observed. Whole-blood gene expression at w0+1d and w1 after i.m. vaccination showed respectively 2611 and 54 significant genes differentially expressed compared with baseline (w0) (**Figure 3A**). With the exception of one outlier, gene expression at baseline was similar between subjects (*data not shown*). The strongest alterations in the blood transcriptome were observed at w0+1d after i.m. immunization (**Figure 3A**). Hierarchical clustering at w0, w0+1d, and w1 showed gene variations at w0+1d (2 611 genes) and w1 (54 genes). The genes significantly upregulated at w0+1d were mainly those involved in the interferon signaling and inflammasome pathways as well as myeloid cells, according to the IPA analysis. The genes significantly downregulated at w0+1d were mainly those involved in EIF2 signaling, a factor that is essential for protein synthesis and regulates proinflammatory cytokine expression pathways (IPA analysis). These genes are also associated with T-cell receptor signalling and iCOS-iCOSL signaling in T helper cells. The IPA analysis highlighted CD4 and CD8+ T cell types (**Figure 3B**).

We also measured inflammatory serum cytokine/chemokine concentrations, including IL-8, CXCL10, IL-1β, TNFα, IL-6, and IL-17α by ultrasensitive Quanterix analyses (**Supplementary Figure 3A**). The nonparametric ANOVA Friedman test showed a significant increase in IL-1β (*p* < 0.001), TNFα (*p* < 0.05) and IL-6 (*p* < 0.005) at 24 h post-vaccination compared to baseline in the i.m. group. In addition, we found that serum levels of CXCL10 were very high in all subjects at w0+1d and w8+1d (maximum detection) (**Supplementary Figure 3A**).

Interestingly, the increased serum level of IL-6 at w0+1d/w0 was significantly correlated with higher MVA-NAb titers at w8 (*p* = 0.014, r = 0.75) (**Figures 3C, D**). No cytokine or chemokine was correlated with the magnitude or polyfunctionality of the MVA-specific CD8 T-cell responses. We therefore focused the further analyses on MVA-NAb responses and hypothesized that the strongest gene signature related to IL-6 might be associated with humoral responses induced by i.m. immunization.

Among the 2611 genes differentially expressed at w0+1d compared with baseline, we identified 124 genes that correlated with MVA-NAb titers at w8 (*p* < 0.05, r > 0.64 and r < -0.64). Among them, 24 genes were involved in the IL-6 pathway, according to the IPA analysis (**Figure 3E**). Most of these genes were overexpressed only at w0+1d and not at w1. Based on the intensity of MVA-NAb responses, we applied hierarchical clustering to the expression profile of the 24 genes related to IL-6 (**Figure 3F**). These genes were found to be involved mainly in antiviral and IFN-signaling, in inflammation, granulocyte maturation, and neutrophil recruitment. Thus, the early IL-6 serum levels and related expressed genes 1 day after vaccination were significantly predictive of the strength of the humoral responses.

### Late Innate Gene Expression at One-Week after t.c. Immunization Correlated With MVA-Specific CD8+ T-Cell Responses

**Figure 4:**
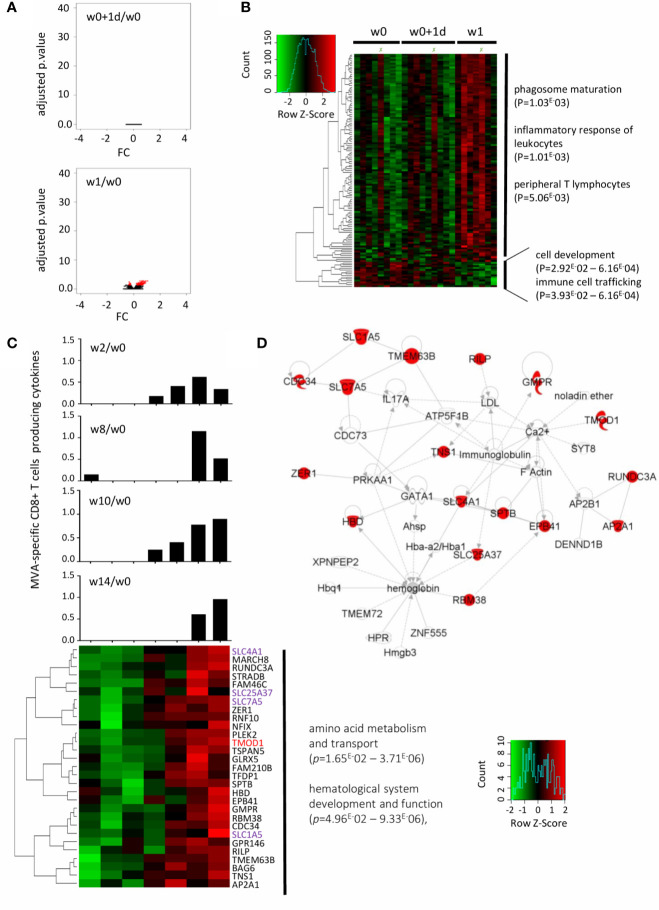
Early gene signature underlying MVA-specific CD8 T-cell responses after t.c. immunization. **(A)** The volcano plot shows no disruption of gene expression at w0+1d (upper panel) and 129 genes significantly differentially expressed at w1 (lower panel) in the t.c. study arm after MVA-B vaccination compared with baseline (w0), according to the -log10(adjusted *p*.value) and log2FC. Significance is defined by an adjusted *p*-value < 0.05 (red). **(B)** The hierarchical clustering is based on the expression profiles of the 129 genes at 3 time points: w0 (n = 8), w0+1d (n = 9), and w1 (n = 7). The color-gradient scale from green (-2, low) to red (2, high) indicates gene expression levels (log2). The functional enrichment was performed with Ingenuity Pathway Analysis software. *p*-values by IPA are indicated for each pathway. **(C)** 29 genes are correlated with the MVA-specific CD8+ T-cell response at w10 determined by the Spearman test with significance based on a *p-*value < 0.05. The hierarchical clustering is performed on the fold-change expression profile (w1/w0) of these 29 genes (lower panel). The genes in common with the literature (24, 25) are in red, and the family of genes in common with it in purple. MVA-specific CD8+ T-cell response intensity at w2, w8, w10, and w14 for each individual are shown (boolean IFNγ, MIP-1β, IL-2, and/or TNFα). **(D)** IPA highest scoring biological network from this minimal gene signature. The highlighted genes (red) are positively correlated with MVA-specific CD8+ T-cell responses.

In contrast to the i.m. study arm, gene expression profiles were not modified at d1 compared with baseline in the t.c. arm (**Figure 4A**, upper panel). However, 129 genes were significantly and differentially expressed compared to baseline at one week after t.c. vaccination (**Figure 4A**, upper panel). These genes are represented by a hierarchical clustering at w0, w0+1d and w1 (**Figure 4B**). The upregulated genes were associated with phagosome maturation, leukocyte inflammatory response, and peripheral T-lymphocyte production, while the genes downregulated at w1 were involved in cell development and in immune cell trafficking (**Figure 4B**). Moreover, we found no elevated cytokines or chemokines induced at w1 after t.c. vaccination (**Supplementary Figure 3B**). Although significant induction of IL-17α occurred, it took place later on (w8 and w9) after t.c. vaccination (**Supplementary Figure 3B**), for an IL-17α level consistent with vaccine induced, MVA-specific CD8+ T-cell responses at around those time points (**Supplementary Figure 4**).

We then sought to define the gene signature at w1 associated with specific CD8+ T-cell responses at these later times. Of the 129 genes significantly disrupted, 29 were correlated with the magnitude of the MVA-specific CD8 T-cells response at w10 (*p* < 0.05, r >0.77). Hierarchical clustering of the expression of these 29 genes at w10 is shown in **Figure 4C**. Histograms showed the percent MVA-specific CD8 T cells at w2, w8, and w14. According to IPA analysis, the biological network related to these genes that scored highest is mainly involved in amino acid metabolism and transport and hematological system development and function (**Figure 4D**).

We also compared the genes correlated with MVA-specific CD8 responses in both the i.m. and t.c. study arm. RILP, for example, was correlated with MVA-specific CD8+ T-cell response in the i.m. study arm (w0+1d) but was also detected at w1 in the t.c. arm. Indeed, in the t.c. arm, RILP was positively correlated with the CD8 T-cell responses at w10 (*p* = 0.025, r = 0.815) and w14 (*p* = 0.040, r = 0.894), and in the i.m. group, with CD8 T-cell responses at w2 (*p* = 0.021, r = 0.743). The RILP gene encodes for the Rab-interacting lysosomal protein associated with the class I MHC-mediated antigen processing and presentation pathway and might thus be a potential biomarker of antigen-specific CD8 responses induced by vaccination by either route of administration. In addition, of the 29 genes modified at w1 in the t.c. arm, several are from the solute carrier (SLC) gene family and were correlated with the magnitude of the MVA-specific CD8 T-cell responses. These membrane transporters are known to be involved in energy homeostasis, metabolic sensing, host defense, oxidative stress, and tissue development.

Overall, the alterations in gene expression profiles induced by t.c. vaccination occurred later and weaklier than those induced by the i.m. route, but were nonetheless sufficient to drive immune responses that were biased toward CD8 T-cell responses.

## Discussion

To our knowledge, CUTHIVAC-003 is the first randomized phase I clinical study comparing administration of an MVA based vaccine by the i.m. and t.c. routes. It is also the first study to analyse the gene expression profile induced by MVA vaccine in relation to both humoral and cell-mediated immune responses. The low samples availability remains the main limitation of the study. For its primary end point, this clinical study showed that the MVA-B vaccine was safe in both groups, although more local and general side effects were observed in the i.m. than in the t.c. group. These results confirm previous reports about the safety of both of these modes of administration (4, 6, 7, 26). We further demonstrated that MVA vaccination by t.c. administration induced only CD8 responses.

To unravel the early events related to the quality of adaptive immune responses, we studied innate and inflammatory molecular mechanisms by measuring whole-blood transcriptomes and serum cytokines at early time points after MVA-B vaccination. We noted that IL-6 and its related genes, all significantly impacted 1 day after i.m. MVA-B vaccination, were correlated with MVA-NAb responses. It has previously been shown that this proinflammatory cytokine is produced *in vitro* and *in vivo* in response to MVA in whole blood, PBMCs, primary human monocytes, and macrophages (27, 28). IL-6 plays an important role in B-cell maturation, is closely associated with antibody production during infections and vaccination, and positively regulates the anti-influenza B-cell responses (24, 29–33). IL-6 is also considered a cytokine adjuvant that enhances immune response to vaccines (34–36). The components of this minimal gene signature related to IL-6 include DHX58, IFIH1, ISG15, and PNPT1, which it shares with the gene signature observed after yellow fever vaccination (37). Other genes that these IL-6 related signatures have in common are CSF3R, NCF4, SAMHD1, and TNFRSF1A, which Nakaya et al. (9) reported to be correlated with the hemagglutination inhibition antibody response after TIV vaccination. Our previous study also showed that TIV immunization induced ISG15 (from IL-6 signalling), while GBP4, FAM111A, and ZBP1 were correlated with neutralizing antibody response (4). All these genes are known to be involved in inflammation, interferon signaling, granulocyte maturation, and neutrophil recruitment.

The comparison of i.m. and t.c. MVA-B delivery routes for innate events demonstrate the earlier and much stronger effect of vaccination in the i.m. compared with the t.c. group. Thus, measured systematically, t.c. vaccination does not adequately induce innate immune responses. Specifically, the only immune responses detected after t.c. immunization were MVA-specific CD8 T-cell responses. Indeed, we found that IL-17α was detected simultaneously with MVA-specific CD8 T-cell responses at late time points. IL-17α is known to be a key proinflammatory cytokine involved in T-cell activation; it is produced by mucosal Th17 lineage and mediates tissue inflammation and memory immune responses (38, 39). We did however report previously that DNA vaccination by a combination of t.c. + i.m. routes compared with the i.d. + i.m. routes shifted responses toward a more Th-17 dominated phenotype (8). Interestingly, in a mice model, LCs are dispensable for the generation of mucosal CTL responses by a mechanism involving IL-17 (40). These cells are the main ones targeted during t.c. vaccination and might thus be involved in the generation of MVA-specific CD8 T-cell responses involving IL-17α. Moreover, the lack of effect by vaccination on either the IL-6 serum concentration in the t.c. group or the IL-17α serum concentration in the i.m. group might support the specificity of these cytokines for each of these routes of administration. This finding might support the specificity of these innate events for respective administration routes.

The SLC family genes correlated with MVA-specific CD8 T-cell responses after t.c. vaccination. Previous studies showed that the amino acid transporters SLC1A5 and SLC7A5 are required for effector T-cell metabolic reprogramming and differentiation after stimulation (25, 41–43). Both of these transporters are also critically important for mediating peripheral naïve T-cell homeostasis, activation, and differentiation, especially for Th1, Th17, and memory T cells (44). This finding might thus explain the indirect relation between IL-17α and SLC7A5 in the top-ranked biological network relating the 29 genes correlated with MVA-specific CD8 T-cell response in the t.c. group. The SLC transporters are also involved in metabolic reprogramming of human monocyte/macrophage immune responses, and serve as metabolic gatekeepers of immune cells (45, 46). Interestingly, numerous SLC family genes are also significantly correlated with cell-mediated responses after yellow fever vaccination (37), although the only shared gene which was correlated with CD8 T-cell response in our study and after yellow fever vaccination was TMOD1 (**Figure 4**) (37). It plays a role in the structure of the erythrocyte membrane skeleton and is involved in TLR and inflammatory signaling modules; it has also been associated with the development of antigen-specific T-cell response to vaccination with MVA85A (47). This further support the notion that the metabolic pathway is the one most relevantly correlated with CD8 T-cell responses, as also observed after influenza and yellow fever vaccinations (4, 37). Moreover, it is interesting to note that the RILP, AP2A1, and CDC34 genes, involved in the class I MHC-mediated antigen processing and presentation pathway, were significantly correlated with MVA-specific CD8+ T cells in the t.c. group. In particular, we found that RILP was common to both routes of immunization and correlated with MVA-specific CD8 T-cell response. RILP plays a major role in protein trafficking in late endosomes. Its reduced expression would suggest that the antigen-presenting capacity of myeloid dendritic cells has been compromised (48, 49).

The limitation of our study is the lack of information on immune cellular profile in the whole blood during vaccination. In order to provide integral immune profile, we have performed an enrichment analysis in order to highlight the cells types based on the impacted genes expressions by vaccination in whole blood (**Supplementary Figure 5**). Based on the 2611 genes significantly differentially expressed 24 h after i.m. immunization the most represented cell types are cells from whole blood (p-value = 3.94E-91), CD33+ myeloid cells (p-value = 5.20E-51), and CD14+ monocytes (p-value = 9.40E-36). And based on the 54 genes significantly differentially expressed 1 week after i.m. immunization the most represented cell types are cells from B lymphoblasts (p-value = 4.29E-5) and CD14+ monocytes (p-value = 0.0037). No gene is found impacted 24 h after t.c. immunization; consequently, no cell type was found, and among the 129 differentially expressed genes 1 week after t.c. immunization, no cell type was found significantly related.

## Conclusion

We have demonstrated that the MVA-B vaccine is safe by both the t.c. and i.m. routes. However, the quality and intensity of both innate and adaptive immunity differ significantly depending on the route of vaccine administration. MVA-B vaccination by the t.c. route promotes CD8 T-cell responses in the absence of humoral response, which results in early biomarkers, apparently especially involved in metabolic pathways, which correlate with late CD8 T-cell responses. On the other hand, MVA-B vaccination by the i.m. route drives important changes in the expression of genes involved in the IL-6 and interferon-signaling pathways, mainly associated with humoral responses. Thus, the vaccine delivery route induces early innate responses that shape the quality of adaptive immunity; this translates into an innate fingerprint that might be used for the development of vaccine formulations that engage particular qualities of the specific immune arms. This work also provides important knowledge about the immunological and transcriptomic impact of MVA in humans, especially useful as this vector has been recently approved by the FDA and EMA for a vaccine against smallpox.

## Data Availability Statement

The original contributions presented in the study are publicly available. This data can be found here: https://www.ebi.ac.uk/arrayexpress/experiments/E-MTAB-9642.

## Ethics Statement

The studies involving human participants were reviewed and approved by The study was registered and approved by the Peru regulatory authorities (IMPACTA IRB 0037-2014-CE; Peru NIH 396-2014-OG-OGITT-OPE/INS). The patients/participants provided their written informed consent to participate in this study.

## Author Contributions

Conceptualization: BC, CB, and JS. Methodology: BC, CB, and JS. Funding: BC, CB, and JS. Acquisition: EG and AL. Validation: SB. Formal analysis: EG, AL, BC, JN, and SB. Investigation: EG, AL, PG, MF, AV, AS, SP, EP, OB, and BM. Supervision: BC, CB, JS, and JL. Resources: BC, CB, JS, JL, CEG, ME, and FG. Data curation: PG, MF, AS, JN, and SB. Writing: EG and BC. Original draft preparation writing: EG and BC. Review editing: All authors. Visualization: EG, BC, and AL. Project administration: BC, CB, and JS. All authors contributed to the article and approved the submitted version.

## Funding

This project has received funding from the European Union’s Seventh Programme for research, technological development and demonstration under grant agreement No 241904, the European Union’s Horizon 2020 Research, Innovation Programme under grant agreement No. 681137, NIH grant P01-AI131568 and La Fondation pour la Recherche Medicale.

## Conflict of Interest

CB is founder, CSO, and shareholder of AELIX THERAPEUTIC. SB is cofounder, CSO and shareholder of AltraBio. JN is employed by AltraBio.

The remaining authors declare that the research was conducted in the absence of any commercial or financial relationships that could be construed as a potential conflict of interest.
