# Supplementary material for: Immune Profiles Identification by Vaccinomics After MVA Immunization in Randomized Clinical Study
Source: Front Immunol. 2020 Nov 10;11:586124. doi: 10.3389/fimmu.2020.586124 (PMC7683801; doi:10.3389/fimmu.2020.586124)
Supplement: Supplementary file 1 [file Presentation_1.pptx]

## Slide 1
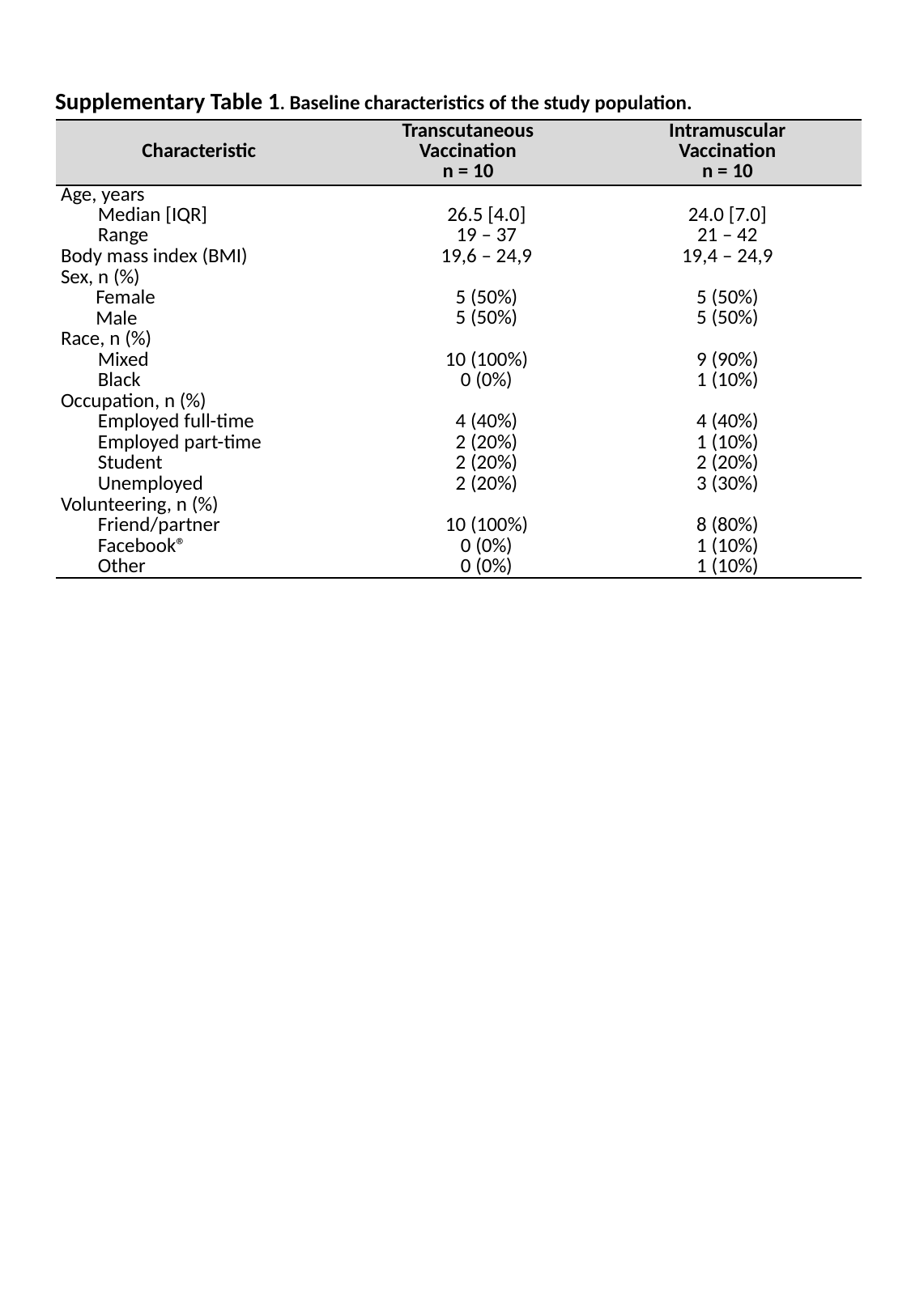

Supplementary Table 1. Baseline characteristics of the study population.
| Characteristic | Transcutaneous Vaccination n = 10 | Intramuscular Vaccination n = 10 |
| --- | --- | --- |
| Age, years | | |
| Median [IQR] | 26.5 [4.0] | 24.0 [7.0] |
| Range | 19 – 37 | 21 – 42 |
| Body mass index (BMI) | 19,6 – 24,9 | 19,4 – 24,9 |
| Sex, n (%) | | |
| Female | 5 (50%) | 5 (50%) |
| Male | 5 (50%) | 5 (50%) |
| Race, n (%) | | |
| Mixed | 10 (100%) | 9 (90%) |
| Black | 0 (0%) | 1 (10%) |
| Occupation, n (%) | | |
| Employed full-time | 4 (40%) | 4 (40%) |
| Employed part-time | 2 (20%) | 1 (10%) |
| Student | 2 (20%) | 2 (20%) |
| Unemployed | 2 (20%) | 3 (30%) |
| Volunteering, n (%) | | |
| Friend/partner | 10 (100%) | 8 (80%) |
| Facebook® | 0 (0%) | 1 (10%) |
| Other | 0 (0%) | 1 (10%) |

## Slide 2
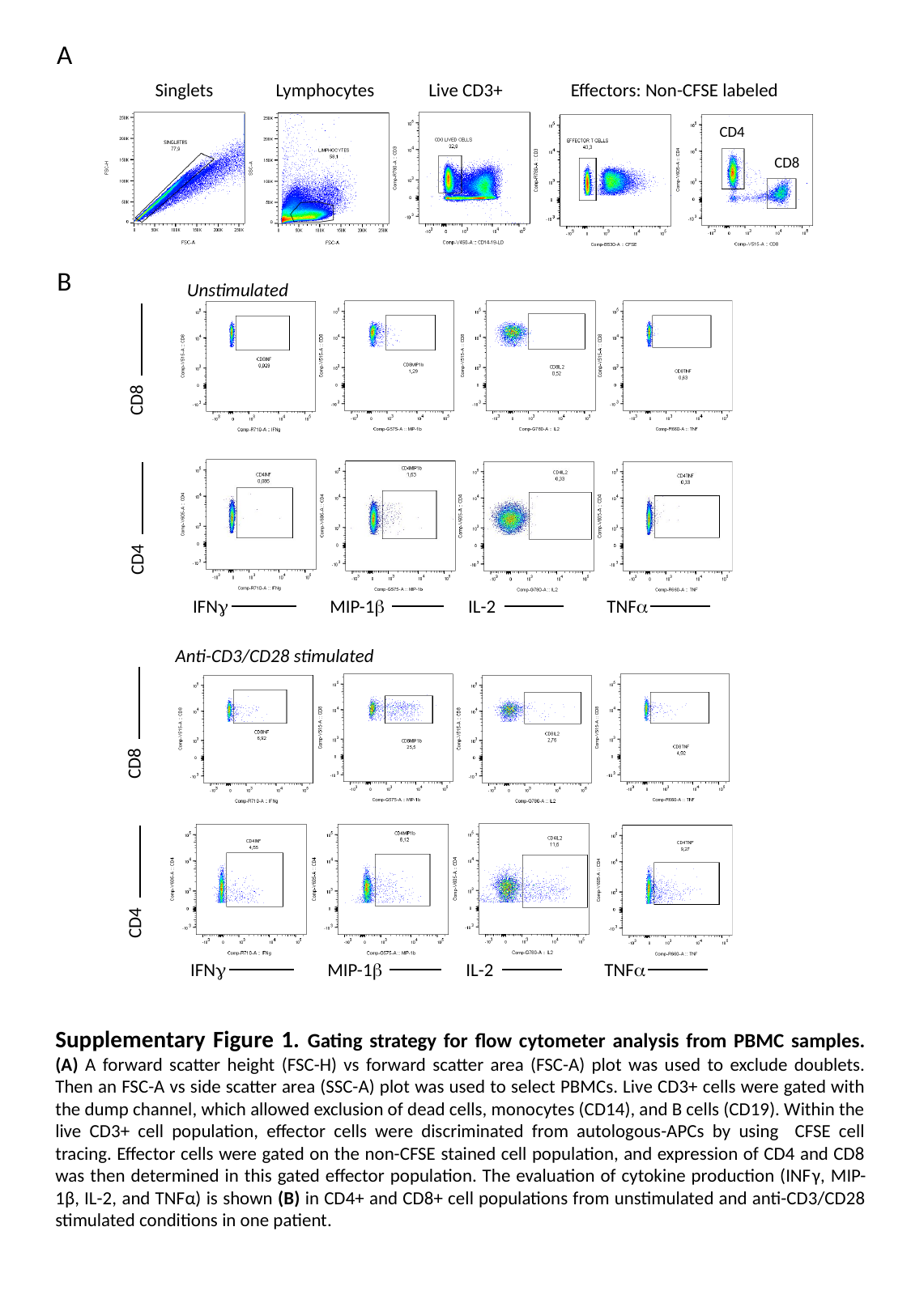

A
Singlets
Lymphocytes
Live CD3+
Effectors: Non-CFSE labeled
CD4
CD8
B
Unstimulated
CD8
CD4
IFNg
MIP-1b
IL-2
TNFa
Anti-CD3/CD28 stimulated
CD8
CD4
IFNg
MIP-1b
IL-2
TNFa
Supplementary Figure 1. Gating strategy for flow cytometer analysis from PBMC samples. (A) A forward scatter height (FSC-H) vs forward scatter area (FSC-A) plot was used to exclude doublets. Then an FSC-A vs side scatter area (SSC-A) plot was used to select PBMCs. Live CD3+ cells were gated with the dump channel, which allowed exclusion of dead cells, monocytes (CD14), and B cells (CD19). Within the live CD3+ cell population, effector cells were discriminated from autologous-APCs by using CFSE cell tracing. Effector cells were gated on the non-CFSE stained cell population, and expression of CD4 and CD8 was then determined in this gated effector population. The evaluation of cytokine production (INFγ, MIP-1β, IL-2, and TNFα) is shown (B) in CD4+ and CD8+ cell populations from unstimulated and anti-CD3/CD28 stimulated conditions in one patient.

## Slide 3
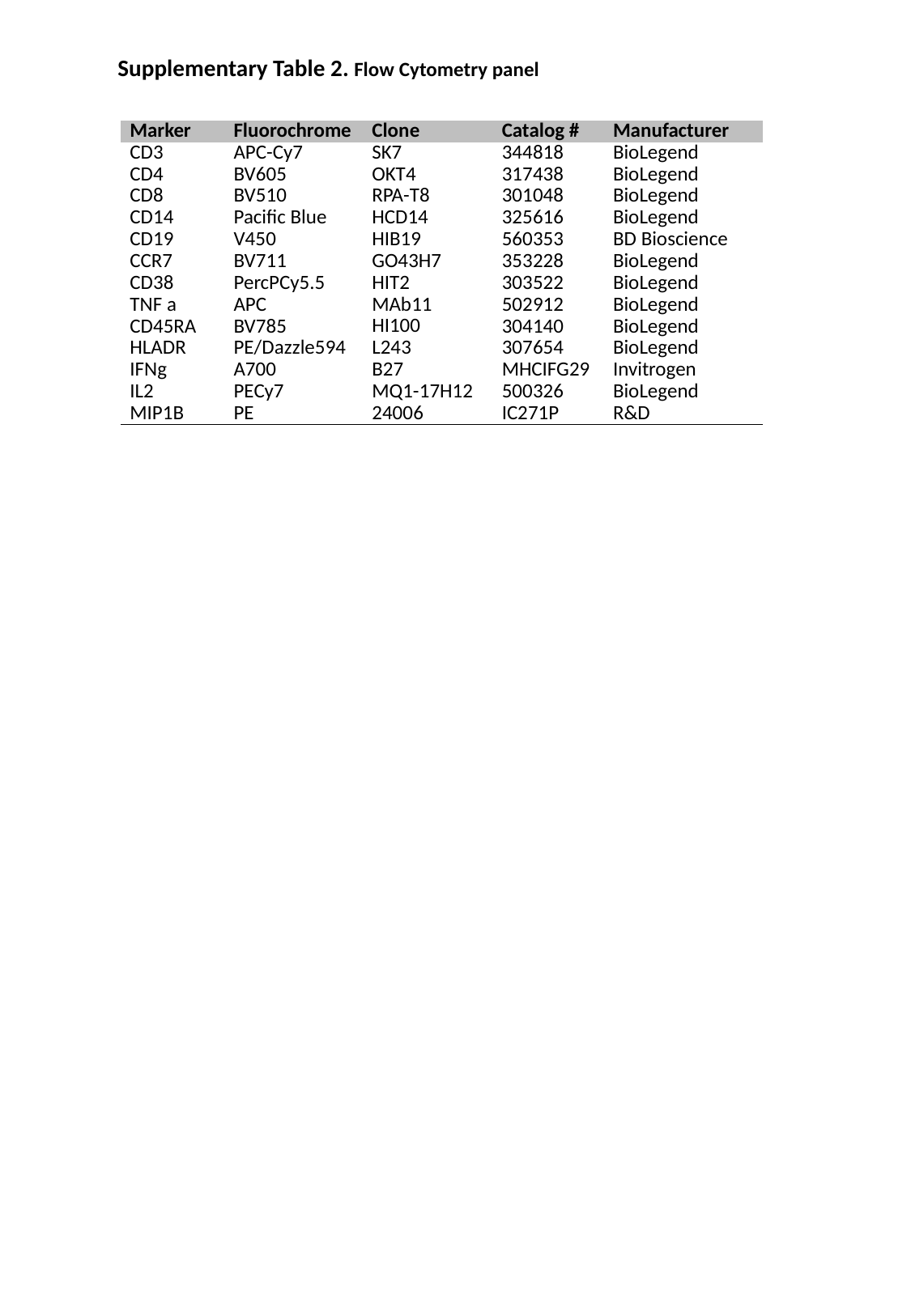

Supplementary Table 2. Flow Cytometry panel
| Marker | Fluorochrome | Clone | Catalog # | Manufacturer |
| --- | --- | --- | --- | --- |
| CD3 | APC-Cy7 | SK7 | 344818 | BioLegend |
| CD4 | BV605 | OKT4 | 317438 | BioLegend |
| CD8 | BV510 | RPA-T8 | 301048 | BioLegend |
| CD14 | Pacific Blue | HCD14 | 325616 | BioLegend |
| CD19 | V450 | HIB19 | 560353 | BD Bioscience |
| CCR7 | BV711 | GO43H7 | 353228 | BioLegend |
| CD38 | PercPCy5.5 | HIT2 | 303522 | BioLegend |
| TNF a | APC | MAb11 | 502912 | BioLegend |
| CD45RA | BV785 | HI100 | 304140 | BioLegend |
| HLADR | PE/Dazzle594 | L243 | 307654 | BioLegend |
| IFNg | A700 | B27 | MHCIFG29 | Invitrogen |
| IL2 | PECy7 | MQ1-17H12 | 500326 | BioLegend |
| MIP1B | PE | 24006 | IC271P | R&D |

## Slide 4
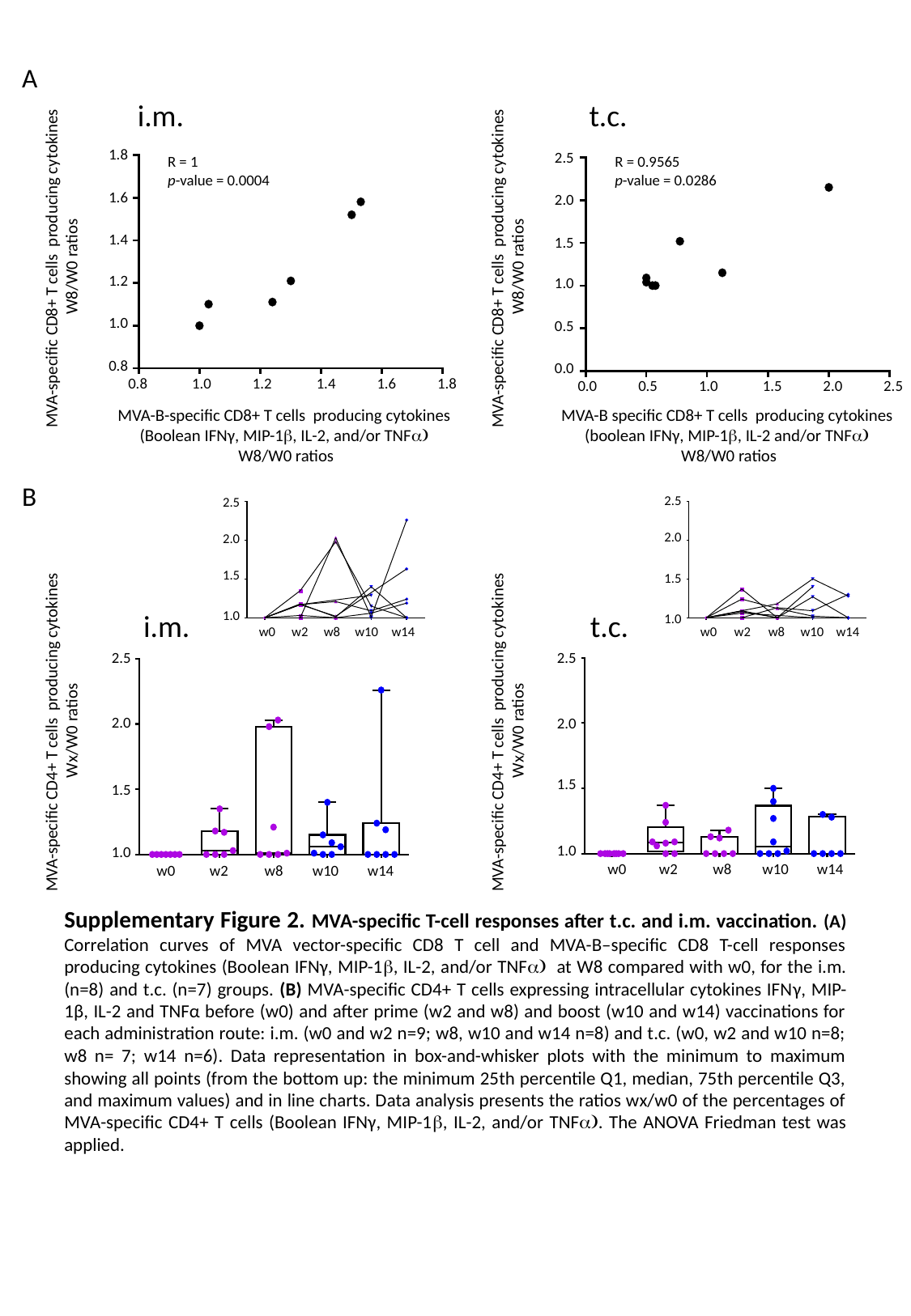

A
i.m.
t.c.
1.8
1.6
1.4
1.2
1.0
0.8
0.8 1.0 1.2 1.4 1.6 1.8
2.5
2.0
1.5
1.0
0.5
0.0
0.0 0.5 1.0 1.5 2.0 2.5
R = 1
p-value = 0.0004
R = 0.9565
p-value = 0.0286
MVA-specific CD8+ T cells producing cytokines
W8/W0 ratios
MVA-specific CD8+ T cells producing cytokines
W8/W0 ratios
MVA-B-specific CD8+ T cells producing cytokines
(Boolean IFNγ, MIP-1b, IL-2, and/or TNFa)
W8/W0 ratios
MVA-B specific CD8+ T cells producing cytokines
(boolean IFNγ, MIP-1b, IL-2 and/or TNFa)
W8/W0 ratios
B
2.5
2.0
1.5
1.0
w0
w2
w8
w10
w14
2.5
2.0
1.5
1.0
w0
w2
w8
w10
w14
i.m.
t.c.
2.5
2.0
1.5
1.0
w0
w2
w8
w10
w14
2.5
2.0
1.5
1.0
w0
w2
w8
w10
w14
MVA-specific CD4+ T cells producing cytokines
Wx/W0 ratios
MVA-specific CD4+ T cells producing cytokines
Wx/W0 ratios
Supplementary Figure 2. MVA-specific T-cell responses after t.c. and i.m. vaccination. (A) Correlation curves of MVA vector-specific CD8 T cell and MVA-B–specific CD8 T-cell responses producing cytokines (Boolean IFNγ, MIP-1b, IL-2, and/or TNFa) at W8 compared with w0, for the i.m. (n=8) and t.c. (n=7) groups. (B) MVA-specific CD4+ T cells expressing intracellular cytokines IFNγ, MIP-1β, IL-2 and TNFα before (w0) and after prime (w2 and w8) and boost (w10 and w14) vaccinations for each administration route: i.m. (w0 and w2 n=9; w8, w10 and w14 n=8) and t.c. (w0, w2 and w10 n=8; w8 n= 7; w14 n=6). Data representation in box-and-whisker plots with the minimum to maximum showing all points (from the bottom up: the minimum 25th percentile Q1, median, 75th percentile Q3, and maximum values) and in line charts. Data analysis presents the ratios wx/w0 of the percentages of MVA-specific CD4+ T cells (Boolean IFNγ, MIP-1b, IL-2, and/or TNFa). The ANOVA Friedman test was applied.

## Slide 5
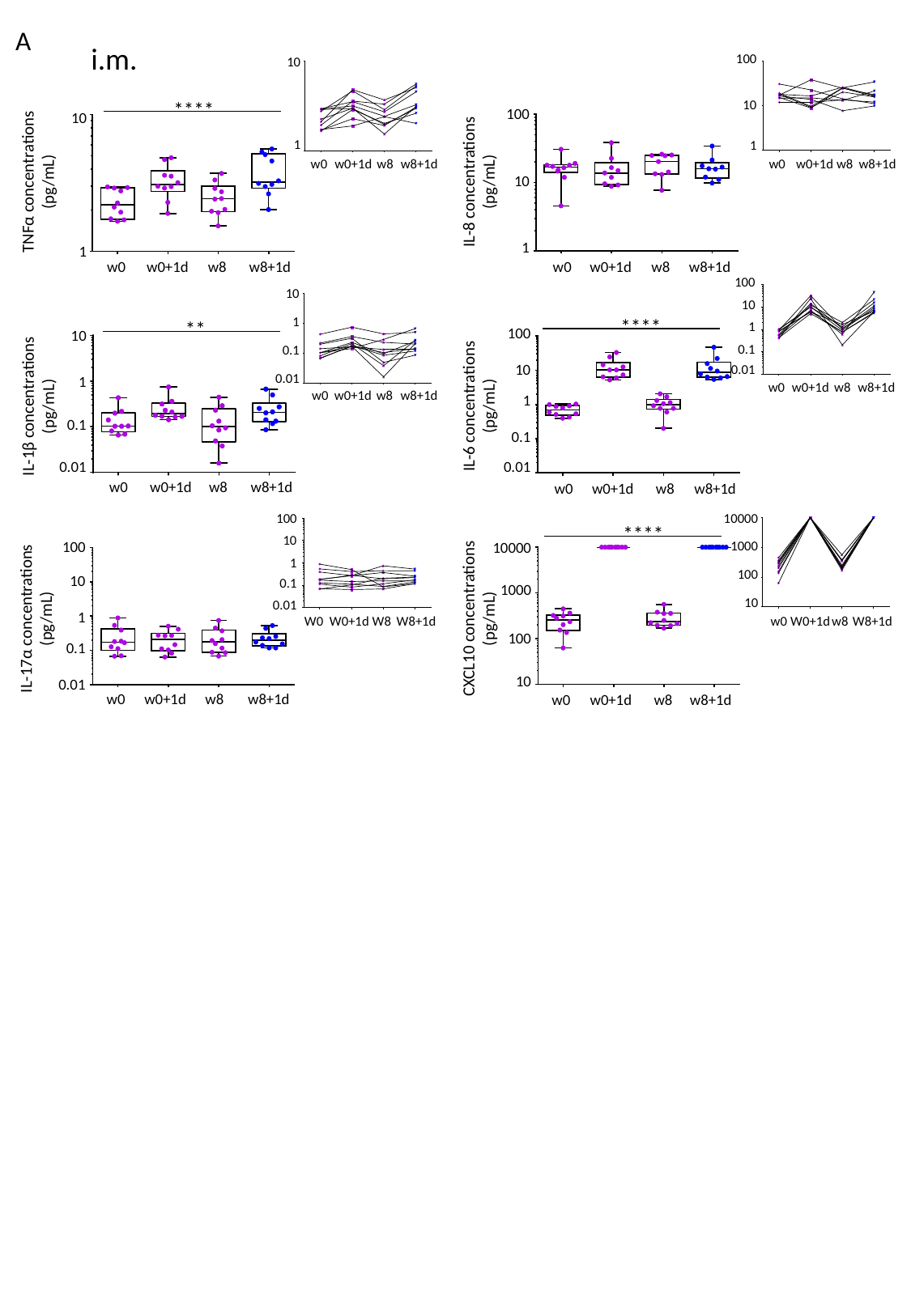

A
100
10
1
w0
w0+1d
w8
w8+1d
10
1
w0
w0+1d
w8
w8+1d
****
100
10
1
w0+1d
w8
w8+1d
w0
10
1
TNFα concentrations
(pg/mL)
IL-8 concentrations
(pg/mL)
w0+1d
w8
w8+1d
w0
i.m.
100
10
1
0.1
0.01
w0
w0+1d
w8
w8+1d
10
1
0.1
0.01
w0
w0+1d
w8
w8+1d
100
10
1
0.1
0.01
****
w0
w0+1d
w8
w8+1d
**
10
1
0.1
0.01
w0
w0+1d
w8
w8+1d
IL-1β concentrations
(pg/mL)
IL-6 concentrations
(pg/mL)
10000
1000
100
10
w0
W0+1d
w8
W8+1d
100
10
1
0.1
0.01
W0
W0+1d
W8
W8+1d
100
10
1
0.1
0.01
w0+1d
w8
w8+1d
w0
10000
1000
100
10
****
IL-17α concentrations
(pg/mL)
CXCL10 concentrations
(pg/mL)
w0
w0+1d
w8
w8+1d

## Slide 6
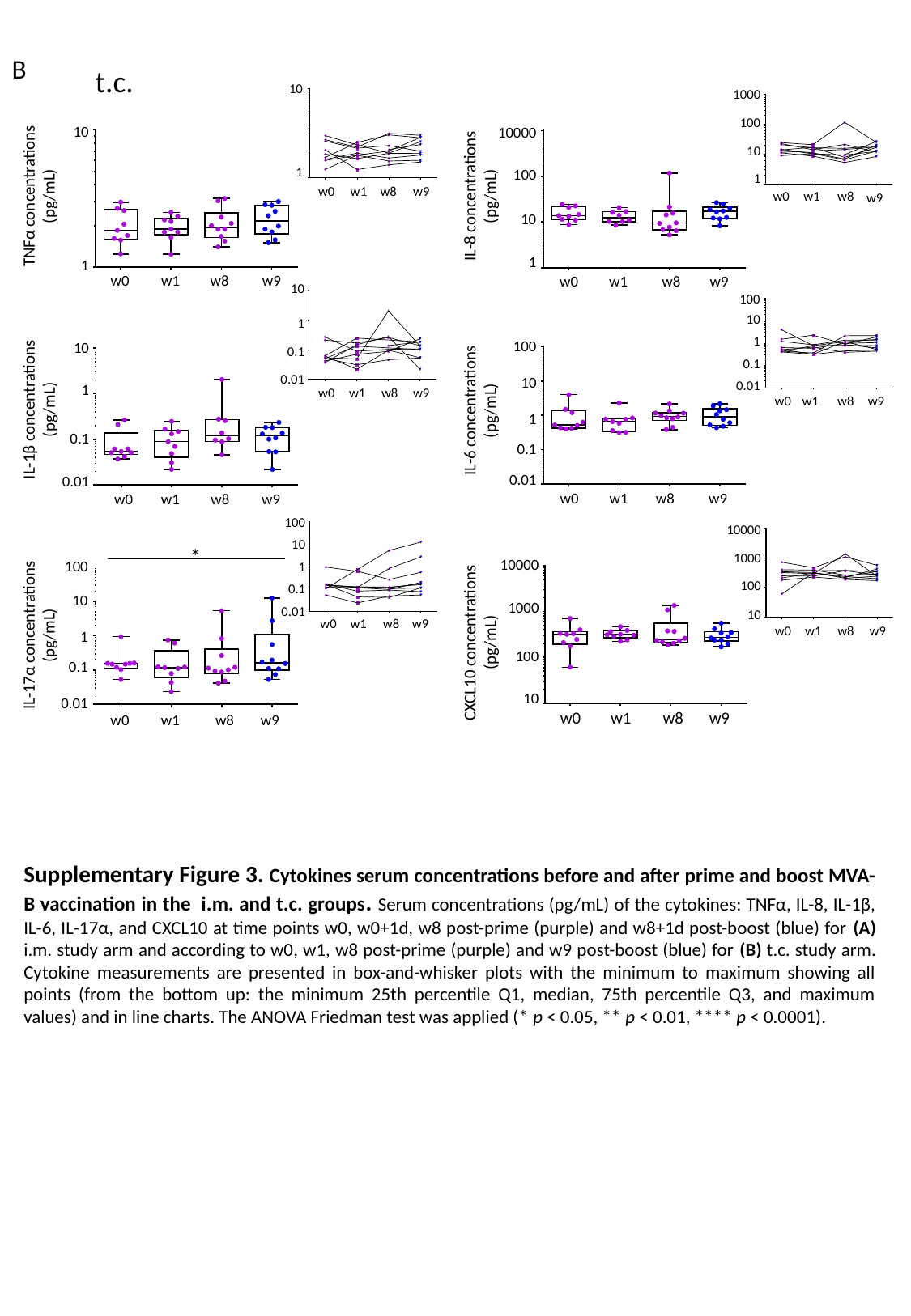

B
t.c.
10
1
w0
w1
w8
w9
1000
100
10
1
w0
w1
w8
w9
10
1
w1
w8
w9
w0
10000
100
10
1
w1
w8
w9
w0
TNFα concentrations
(pg/mL)
IL-8 concentrations
(pg/mL)
10
1
0.1
0.01
w0
w1
w8
w9
100
10
1
0.1
0.01
w0
w1
w8
w9
100
10
1
0.1
0.01
w8
w0
w1
w9
10
1
0.1
0.01
w1
w8
w9
w0
IL-1β concentrations
(pg/mL)
IL-6 concentrations
(pg/mL)
100
10
1
0.1
0.01
w0
w1
w8
w9
10000
1000
100
10
w0
w1
w8
w9
10000
1000
100
10
100
10
1
0.1
0.01
*
w0
w1
w8
w9
w0
w1
w8
w9
IL-17α concentrations
(pg/mL)
CXCL10 concentrations
(pg/mL)
Supplementary Figure 3. Cytokines serum concentrations before and after prime and boost MVA-B vaccination in the i.m. and t.c. groups. Serum concentrations (pg/mL) of the cytokines: TNFα, IL-8, IL-1β, IL-6, IL-17α, and CXCL10 at time points w0, w0+1d, w8 post-prime (purple) and w8+1d post-boost (blue) for (A) i.m. study arm and according to w0, w1, w8 post-prime (purple) and w9 post-boost (blue) for (B) t.c. study arm. Cytokine measurements are presented in box-and-whisker plots with the minimum to maximum showing all points (from the bottom up: the minimum 25th percentile Q1, median, 75th percentile Q3, and maximum values) and in line charts. The ANOVA Friedman test was applied (* p < 0.05, ** p < 0.01, **** p < 0.0001).

## Slide 7
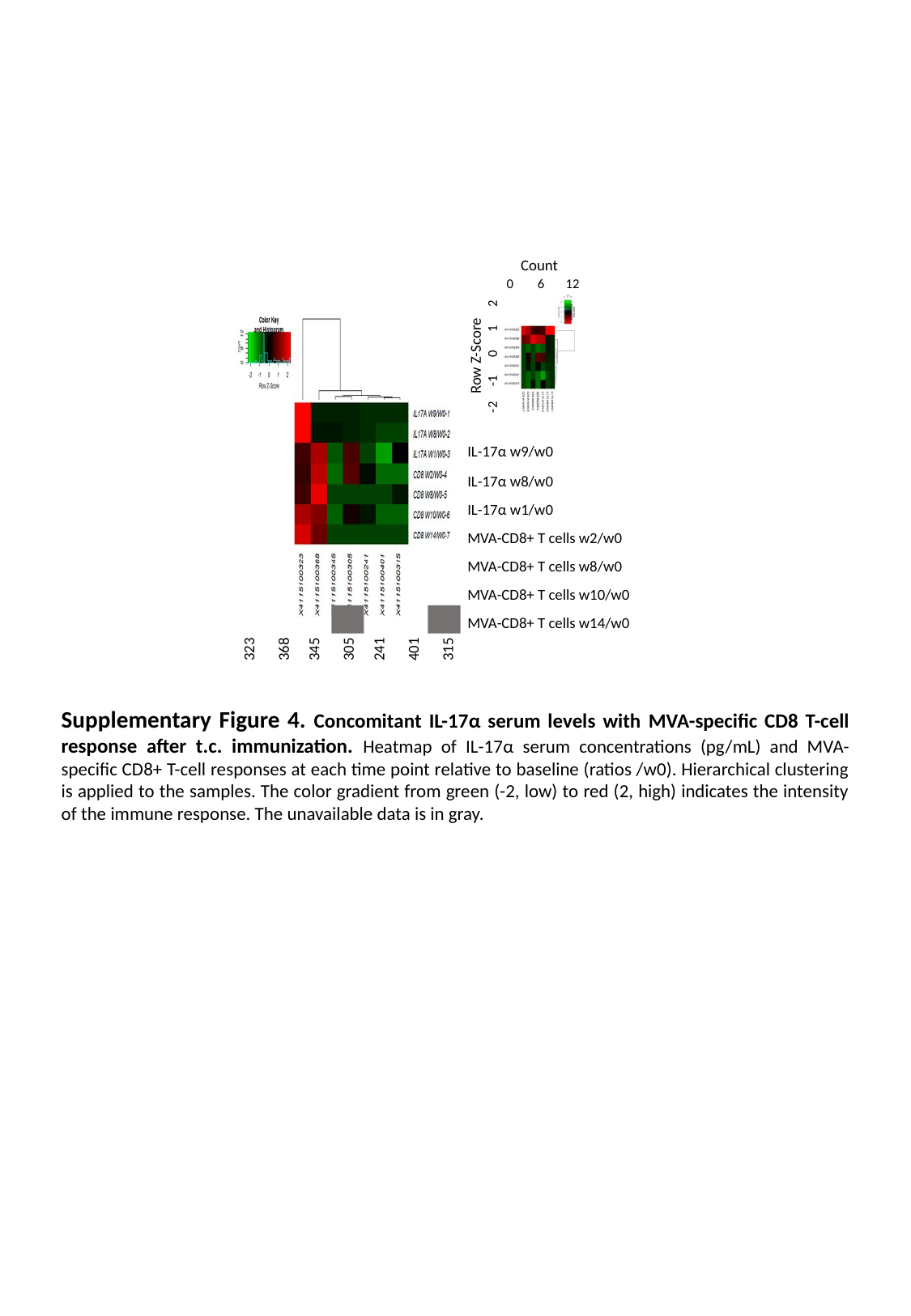

Count
-2 -1 0 1 2
Row Z-Score
0 6 12
IL-17α w9/w0
IL-17α w8/w0
IL-17α w1/w0
MVA-CD8+ T cells w2/w0
MVA-CD8+ T cells w8/w0
MVA-CD8+ T cells w10/w0
MVA-CD8+ T cells w14/w0
323
368
345
305
241
401
315
Supplementary Figure 4. Concomitant IL-17α serum levels with MVA-specific CD8 T-cell response after t.c. immunization. Heatmap of IL-17α serum concentrations (pg/mL) and MVA-specific CD8+ T-cell responses at each time point relative to baseline (ratios /w0). Hierarchical clustering is applied to the samples. The color gradient from green (-2, low) to red (2, high) indicates the intensity of the immune response. The unavailable data is in gray.

## Slide 8
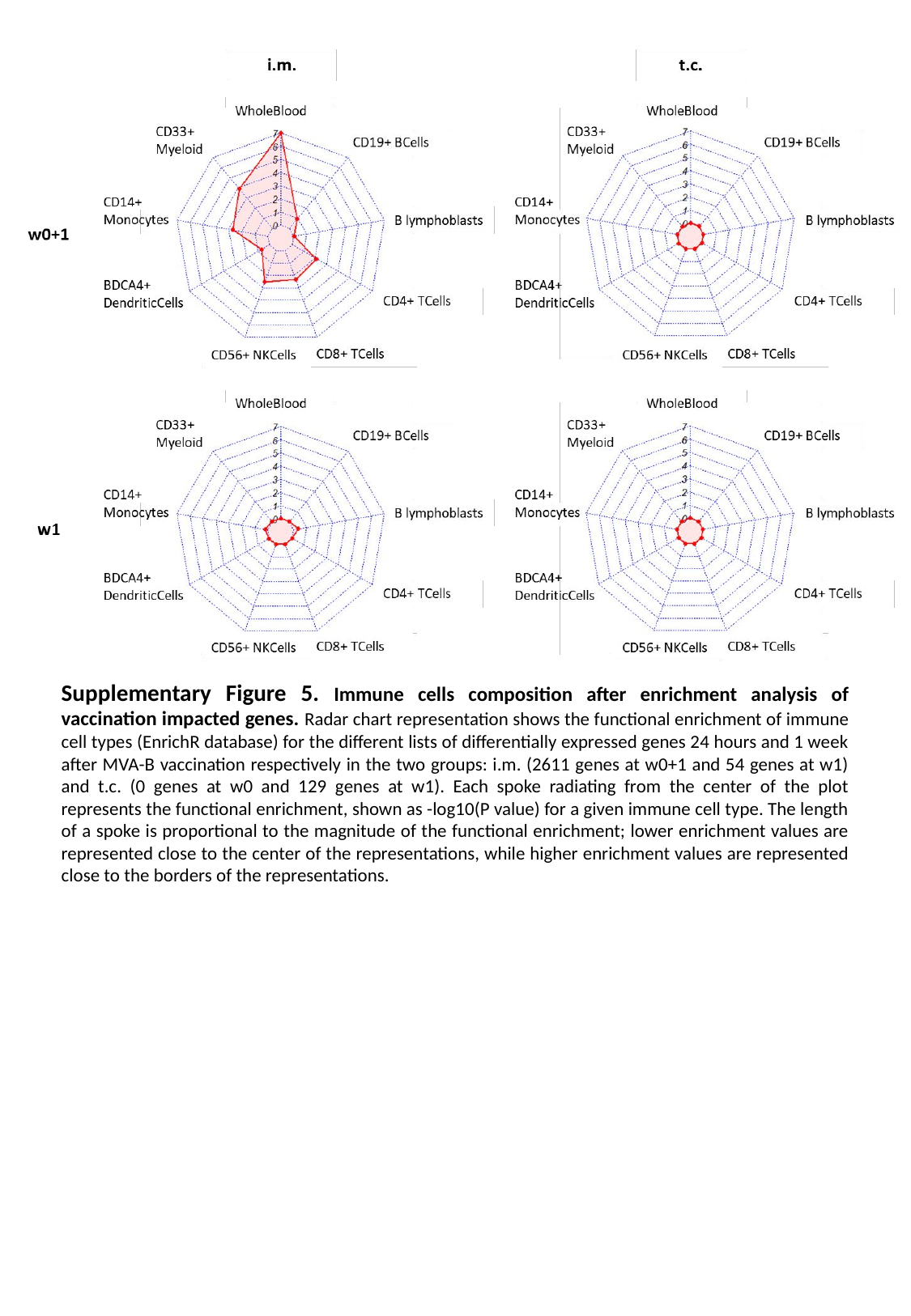

Supplementary Figure 5. Immune cells composition after enrichment analysis of vaccination impacted genes. Radar chart representation shows the functional enrichment of immune cell types (EnrichR database) for the different lists of differentially expressed genes 24 hours and 1 week after MVA-B vaccination respectively in the two groups: i.m. (2611 genes at w0+1 and 54 genes at w1) and t.c. (0 genes at w0 and 129 genes at w1). Each spoke radiating from the center of the plot represents the functional enrichment, shown as -log10(P value) for a given immune cell type. The length of a spoke is proportional to the magnitude of the functional enrichment; lower enrichment values are represented close to the center of the representations, while higher enrichment values are represented close to the borders of the representations.
